# Association between the blood pressure variability and cognitive decline in Parkinson's disease

**DOI:** 10.1002/brb3.3319

**Published:** 2023-11-15

**Authors:** Yi Xiao, Tianmi Yang, Lingyu Zhang, Qianqian Wei, Ruwei Ou, Yanbing Hou, Kuncheng Liu, Junyu Lin, Qirui Jiang, Huifang Shang

**Affiliations:** ^1^ Department of Neurology Rare Disease Center, Laboratory of Neurodegenerative Disorders, National Clinical Research Center for Geriatric West China Hospital of Sichuan University Chengdu Sichuan China; ^2^ Health Management Center West China Hospital of Sichuan University Chengdu China

**Keywords:** ambulatory blood pressure monitoring, biomarker, cognition, Parkinson's disease

## Abstract

**Objectives:**

High visit‐to‐visit blood pressure variability (BPV) was found to be associated with cognitive decline in the elderly. This study aimed to investigate the impact of visit‐to‐visit BPV on cognition in patients with early‐stage Parkinson's disease (PD).

**Design:**

This is a retrospective analysis of a prospective cohort.

**Setting and participants:**

A total of 297 patients with early‐stage PD (103 mild cognitive impairments [PD‐MCI] and 194 normal cognitions [PD‐NC] at baseline) were included from the Parkinson's Progression Markers Initiative study.

**Methods:**

Variation independent of mean (VIM) of the first year was used as the indicator of BPV. The Montreal Cognitive Assessment (MoCA) was used to assess global cognition. Patients were divided into PD‐MCI and PD‐NC according to the MoCA score at baseline. Longitudinal cerebrospinal fluid (Aβ‐42, Aβ, α‐synuclein, neurofilament light protein, tau phosphorylated at the threonine 181 position, total tau, glial fibrillary acidic protein) and serum (neurofilament light protein) biomarkers were assessed. The Bayesian linear growth model was used to evaluate the relationship between baseline BPV and the rate of change in cognition and biomarkers.

**Results:**

Higher systolic VIM of the first year was related to a greater rate of decline in MoCA score in the following years in PD‐MCI (*β* = −.15 [95% CI −.29, −.01]). No association was found between BPV and biomarkers.

**Conclusion and implications:**

Higher systolic VIM predicted a steeper decline in cognitive tests in PD‐MCI independently from the mean value of blood pressure, orthostatic hypotension, and supine hypertension.

## Graphical abstract

Higher systolic visit‐to‐visit blood pressure variability (BPV) was related to a greater rate of decline in the score of Montreal Cognitive Assessment in the follow‐up in patients with Parkinson's disease with mild cognitive impairments (β = −.15 [95% CI −.29, −.01]). No association was found between BPV and cognition in patients with Parkinson's disease with normal cognition.

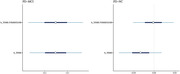

## 1 INTRODUCTION

Dementia is one of the most devastating symptoms of Parkinson's disease (PD) (Svenningsson et al., 2012). Recent studies proposed the prognostic values of visit‐to‐visit blood pressure variability (BPV) in the field of cognition (de Heus et al., 2019; Rouch et al., 2020). Higher BPV predicted dementia in older people (Rouch et al., 2020). Alzheimer's disease patients with higher BPV had a faster cognitive decline than those with lower BPV after a 1‐year follow‐up (de Heus et al., 2019). Previous studies found that BP abnormalities such as orthostatic hypotension (OH) and supine hypertension (SH) were associated with a higher risk of dementia in the follow‐up in PD (Anang et al., 2014; Longardner et al., 2020). One study found that patients with PD with mild cognitive impairment (PD‐MCI) had a significantly higher diastolic BPV during follow‐up than those with normal cognition (PD‐NC), indicating that the BPV was a potential predictive marker of cognitive impairment (Kwon et al., 2016). However, there is a lack of research on the association between BPV and the longitudinal cognitive change in PD.

In the current study, we aimed to figure out the relationship between visit‐to‐visit supine BPV and the progression of cognitive decline in patients with early‐stage PD. As previous studies indicated that the increasing BPV might be a result of the neurodegenerative process, we divided patients into PD‐MCI and PD‐NC to exclude the potential reverse causality.

## 2 METHODS

### 2.1 Patients

The data was downloaded from the website of PPMI (www.ppmi‐info.org) on December 28, 2021. The inclusion criteria of the current study were (1) disease duration ≤3 years; (2) having at least three records of BP from baseline to 12‐month follow‐up; (3) having at least one follow‐up record of cognitive test after baseline; and (4) without dementia (Montreal Cognitive Assessment [MoCA] score ≥21) at baseline (Dalrymple‐Alford et al., 2010). The exclusion criteria were patients who had missing data at baseline. Patients were divided into PD‐NC or PD‐MCI according to the level I diagnostic criteria of PD‐MCI (Litvan et al., 2012). PD‐MCI was defined as a MoCA score ≤26 and without daily activity deficit caused by cognitive decline (Hoops et al., 2009). Written informed consent was obtained from all participants.

### 2.2 Evaluation

Experienced neurological doctors conducted face‐to‐face evaluations with patients. BP was recorded after lying down for 1−3 min on the examination bed and at 1−3 min after standing up from the bed. OH was defined as a drop of systolic BP ≥20 mL of mercury (mmHg) and/or diastolic BP ≥10 mmHg. SH was defined as supine systolic BP ≥140 mmHg or supine diastolic BP ≥90 mmHg (Fanciulli et al., 2018). Variation independent of the mean (VIM) was not related to the mean blood pressure value, and VIM of the first year of BP was used as the BPV indicator in the current study (Asayama et al., 2015). The Supporting Information section described the details of the calculation of the VIM. The MoCA assessed the global cognition situation. The Movement Disorder Society‐sponsored revision of the Unified Parkinson's Disease Rating Scale (MDS‐UPDRS) was used to evaluate the disease severity. The rapid eye movement sleep behavior disorder was evaluated using the rapid eye movement sleep behavior disorder screening questionnaire. Depression was evaluated using the geriatric depression scale (Yesavage et al., 1982). The apolipoprotein E genotyping (ε2, ε3, and *ε*4 alleles) was determined, and patients were divided into ε4 carriers or non‐ε4 carriers.

The following Alzheimer's disease‐related biomarkers and PD‐related biomarkers were collected: cerebrospinal fluid (CSF) amyloid‐β1‐42, CSF Aβ, CSF α‐synuclein, serum, and CSF neurofilament light protein, CSF tau phosphorylated at the threonine 181 position, CSF total tau, and CSF glial fibrillary acidic protein. The details of the biomarker assessment and quality control were displayed on the website of PPMI (www.ppmi‐info.org).

### 2.3 Data analyses

Median (quartile) and frequency (percent) were used to describe the quantitative and qualitative data. The distribution was tested with Shapiro–Wilk tests, and the data was found to be not normally distributed. The two‐sided Mann–Whitney *U* test, chi‐square test, or Fisher's exact test were used to compare the demographic characteristics of patients between groups as appropriate.

Bayesian linear growth model was conducted using the R package *brms*. Interaction terms between the time and BPV indicators were used to explore the association of first year BPV and the rate of change in cognitive test scores and biomarkers in the following years. Random intercept and random slope were set for the individual. Models were adjusted for sex, education, history of comorbidities (hypertension, hyperlipidemia, and heart disease), use of the antihypertension drug, apolipoprotein E status, clinical characteristics at the first year assessment (disease duration, age, MDS‐UPDRS part III score, MoCA score, OH, SH, rapid eye movement sleep behavior disorder screening questionnaire score, and geriatric depression scale score), and means of first year BP depending on the BPV indicators. The results of the Bayesian linear growth models are displayed as β and the 95% confidence interval (95% CI). Statistical significance was indicated as the 95% CI did not include zero. We further did two sensitivity analyses adjusted for anti‐Parkinson drugs and applied alternative diagnostic criteria (see Supporting Information section). IBM SPSS version 22.0 and R version 4.1.2 were used for the statistical analysis, and all the tests were two‐sided.

## 3 RESULTS

### 3.1 Impacts of supine BPV on MoCA and biomarkers

**FIGURE 1:**
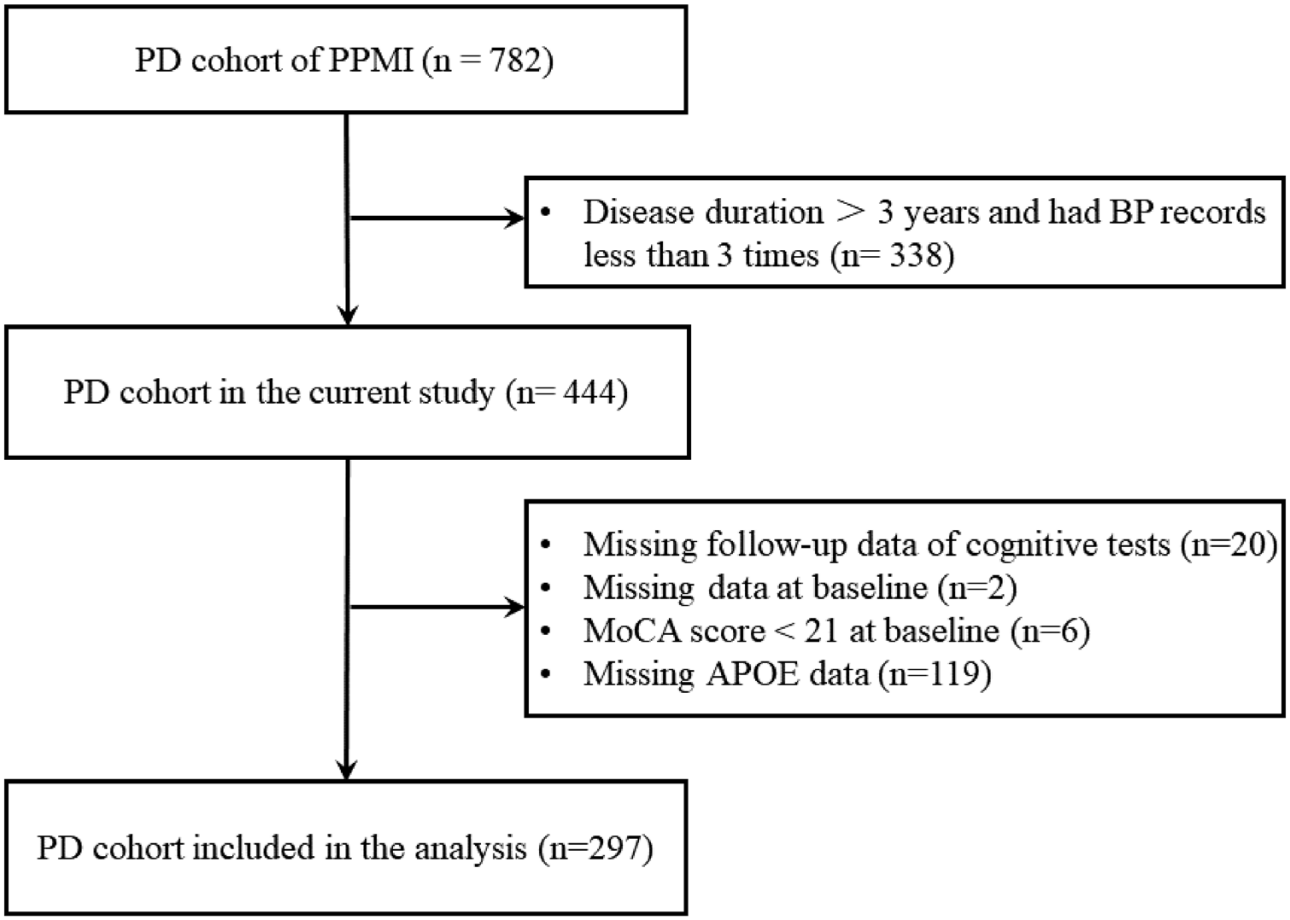
Flow diagram of the number of patients included and excluded from the visit‐to‐visit blood pressure variability analysis. BP, blood pressure; MoCA, Montreal Cognitive Assessment; PD, Parkinson's disease; PPMI, Parkinson's Progression Markers Initiative.

**FIGURE 2:**
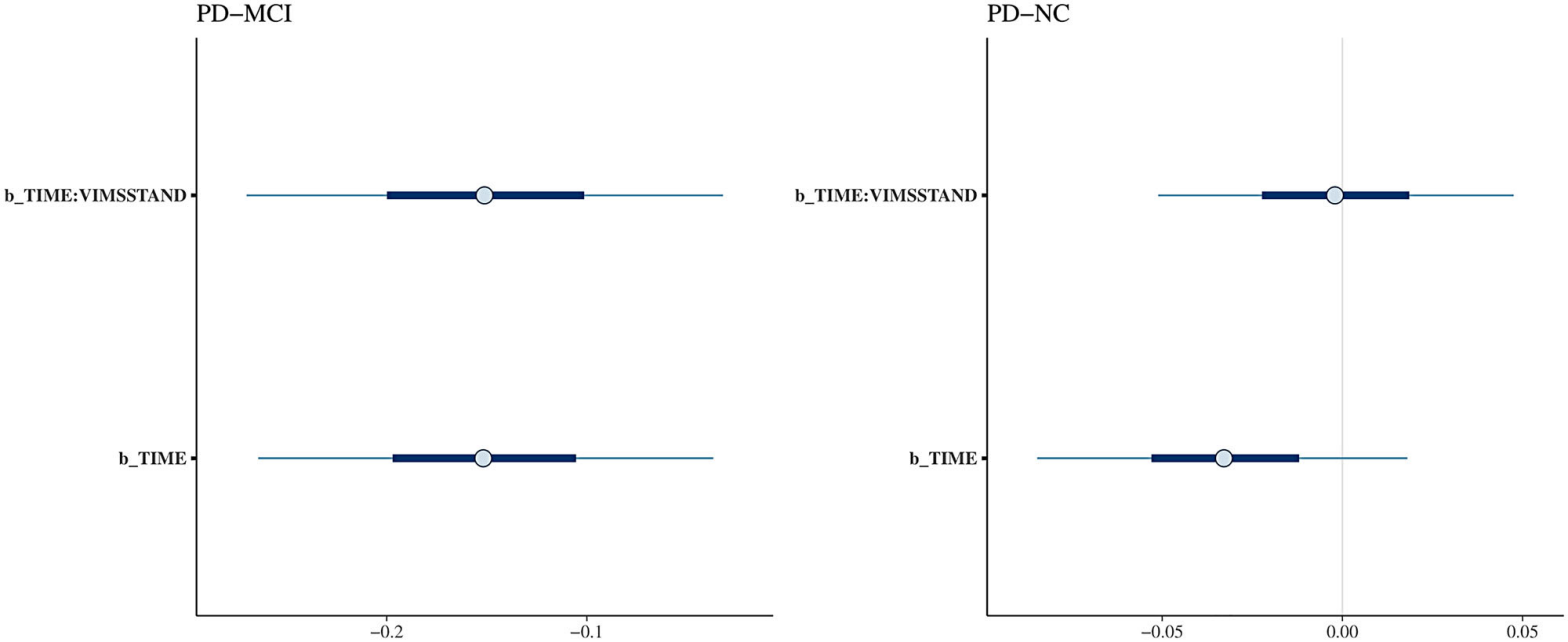
Impact of time and systolic variation independent of mean (VIM) on cognition in PD‐NC and PD‐MCI. The β and 95%CI of time and interaction of time and systolic variation independent of the mean was displayed. MCI, mild cognitive impairment; NC, normal cognition; PD, Parkinson's disease.

A total of 297 patients ended up being included in the study, with 103 PD‐MCI and 194 PD‐NC (Figure 1). Demographic characteristics are shown in Table 1. There were no differences in VIM among patients with or without OH, patients with or without hypertension, and patients with or without SH (Table S1). The results of the impacts of BPV on MoCA and biomarkers in PD‐MCI and PD‐NC are displayed in Table 2. The average times of follow‐up of MoCA and biomarkers were listed in Table S2. In PD‐MCI, higher systolic VIM in the first year was associated with a steeper decrease in MoCA score in the following years (*β* = −0.15 [95% CI −0.29, −.01]) (Figure 2). The diastolic VIM was not associated with the rate of the MoCA score. Both the systolic and the diastolic VIM were not associated with the rate of the biomarkers. In PD‐NC, there were no associations between the first year VIM and the MoCA score or biomarkers in the following years.

**TABLE 1:** Characteristics of patients with Parkinson's disease at baseline.

Characteristics median (quartile)	All patients (*N* = 297)	PD‐MCI (*n* = 103)	PD‐NC (*n* = 194)	*p* Value
Male, *n* (%)	202 (68.0%)	74 (71.8%)	128 (66%)	.302
Age (year)	62.1 (54.8–68.55)	64.5(57.5–69.7)	60.75 (52.43–67.7)	<.001^a^
Disease duration (year)	1.08 (.75–1.79)	1.08 (.75–1.83)	1.08 (.75–1.75)	.650
Education (year)	16 (14–18)	16 (14–18)	16 (14–17.25)	.856
BMI (kg/m^2^)	26.42 (24.05–−29.65)	26.37 (24.04–29.56)	26.47 (24.05–29.9)	.663
MDS‐UPDRS part III score	21 (15–26)	23 (18–29)	18 (13–26)	<.001^a^
RBDSQ score	5 (3–6.5)	5 (3–7)	4 (3–6)	.415
GDS score	2 (1–3)	2 (1–3)	2 (1–3)	.549
MOCA score	28 (26–29)	25 (24–26)	28 (28–29)	<.001^a^
*APOE* ε4 carrier	77 (25.9%)	22 (21.4%)	55 (28.4%)	.191
Systolic blood pressure variability				
Blood pressure (mmHg)	130 (120–141)	133 (122–141)	130 (120–141)	.375
Mean (mmHg)	129.6 (122.17–139)	131.5 (122.8–140.25)	128.4 (120.95–137.75)	.125
VIM (mmHg)	8.03 (5.71–10.5)	8.25 (6.26–10.78)	7.79 (5.42–10.49)	.601
Diastolic blood pressure variability				
Blood pressure (mmHg)	79 (71–85)	78 (70–84)	80 (72–86)	.236
Mean (mmHg)	78 (72.6–83.33)	77.8 (72.5–82.5)	78.27 (73.05–83.85)	.382
VIM (mmHg)	5.4 (3.78–7.34)	5.79 (3.78–7.31)	5.16 (3.75–7.39)	.501
OH, *n* (%)	42 (14.1%)	21 (20.4%)	21 (10.8%)	.024^a^
SH, *n* (%)	107 (36%)	38 (36.9%)	69 (35.6%)	.821
Medical history, *n* (%)				
Hypertension	119 (40.1%)	50 (48.5%)	69 (35.6%)	.030^a^
Dyslipidemia	27 (9.1%)	13 (12.6%)	14 (7.2%)	.123
Type 2 diabetes mellitus	2 (.7%)	1 (1%)	1 (.5%)	1.000
Heart disease	11 (3.7%)	3 (2.9%)	8 (4.1%)	.753
Use of antihypertension drug, *n* (%)	116 (39.1%)	49 (47.6%)	67 (34.5%)	.028^a^

Abbreviations: BMI, body mass index; GDS, geriatric depression scale; MCI, mild cognitive impairment; MDS‐UPDRS, The Movement Disorder Society‐sponsored revision of the Unified Parkinson's Disease Rating Scale; MoCA, Montreal Cognitive Assessment; NC, normal cognition; OH, orthostatic hypotension; PD, Parkinson's disease; RBDSQ, rapid eye movement sleep behavior disorder screening questionnaire; SH, supine hypertension; VIM, variation independent of the mean.

^a^ Significant at level 0.05.

**TABLE 2:** Impact of first year variation independent of mean (VIM) on the rate of change in outcomes (TIME × VIM).

	PD‐MCI		PD‐NC	
Outcomes	*β*	95%CI	*β*	95%CI
Systolic VIM				
MoCA	−.15	−.29 to −.01^a^	−.00	−.06 to .06
CSF Aβ42	−4.87	−38.94 to 29.53	−6.27	−36.35 to 24.20
CSF Aβ	.25	−27.10 to 27.10	5.16	−10.90 to 21.52
Serum Nfl	.21	−.32 to .75	.25	−.24 to .75
CSF NfL	2.93	−10.97 to 15.66	−.61	−3.26 to 2.02
CSF ptau‐181	.11	−.13 to .35	−.08	−.22 to .06
CSF total tau	1.28	−1.65 to 4.17	−1.45	−3.23 to .27
CSF α‐synuclein	−47.04	−104.86 to 10.93	−6.23	−43.14 to 30.87
CSF GFAP	.08	−.25 to .41	−.11	−.29 to .09
Diastolic VIM				
MoCA	−.08	−.24 to .08	.02	−.03 to .08
CSF Aβ42	5.73	−34.62 to 45.87	−4.77	−30.63 to 21.32
CSF Aβ	29.27	−1.90 to 60.51	−1.84	−14.49 to 10.75
Serum Nfl	.07	−.54 to .69	−.12	−.59 to .34
CSF NfL	3.07	−11.49 to 17.57	1.69	−.13 to 3.53
CSF ptau‐181	.14	−.13 to .41	−.03	−.14 to .08
CSF total tau	2.51	−.72 to 5.73	−.68	−2.13 to .76
CSF α‐synuclein	12.67	−56.98 to 82.13	2.24	−28.27 to 31.86
CSF GFAP	.10	−.24 to .44	.06	−.06 to .19

Abbreviations: Aβ, β‐amyloid; Aβ42, amyloid‐β1‐42; CSF, cerebrospinal fluid; GFAP, glial fibrillary acidic protein; MCI, mild cognitive impairment; MoCA, Montreal Cognitive Assessment; NC, normal cognition; NfL, neurofilament light protein; p‐tau181, tau phosphorylated at the threonine 181 position; VIM, variation independent of the mean.

^a^ 95% CI not include 0.

### 3.2 Sensitivity analysis

Similar results were reached when implementing the sensitivity analysis by including the time‐varying levodopa equivalent drug dose, use of dopamine agonists, amantadine, monoamine oxidase‐*B* inhibitors, and antidementia agents in the models (data not shown). Higher systolic VIM was associated with a faster decrease in MoCA score in PD‐MCI (*β* = −.14 [95% CI −.29, −.01]). Applying the alternative diagnostic criteria of PD‐MCI also showed the same results (data not shown). There were 44 patients diagnosed with PD‐MCI, and higher systolic VIM was still related to a faster deterioration in MoCA score in PD‐MCI (*β* = −.31 [95% CI −.57, −.06]).

## 4 DISCUSSION

In this longitudinal cohort study, the relationship between supine position BPV and cognitive changes in 297 patients with PD was explored. The key finding revealed that elevated systolic VIM was associated with a faster decline in global cognitive function, as assessed by MoCA, in patients with PD‐MCI. Similar observations have been reported in studies involving older individuals and patients with Alzheimer's disease (Böhm et al., 2015; Ernst et al., 2021; Lattanzi et al., 2015; Qin et al., 2016; Rouch et al., 2020). Chinese older individuals with high systolic BPV experienced a more rapid cognitive decline compared to those with low systolic BPV (Qin et al., 2016). Patients with chronic diseases who exhibited elevated systolic BPV had a higher risk of developing incident dementia (Rouch et al., 2020).

The underlying mechanism connecting BPV and cognition is still under investigation. Increased BPV might stem from the advancement of neurodegeneration. The central autonomic network includes the insular cortex, anterior cingulate cortex, and amygdala (Cersosimo & Benarroch, 2013). Dysfunction in these areas has also been observed in patients with PD experiencing cognitive impairments (Christopher et al., 2014; de la Monte et al., 1989; Kalaitzakis et al., 2009). Additionally, behavioral changes resulting from cognitive impairment may contribute to elevated BPV. For example, patients with cognitive impairment often exhibit poorer medication management and treatment adherence compared to cognitively normal patients. This discrepancy leads to greater fluctuations in blood pressure among patients with PD who also have hypertension (Sumbul‐Sekerci et al., 2022).

A contrary hypothesis has also been proposed suggesting that high BPV leads to reduced cognition. Elevated systolic BPV has been linked to increased arterial stiffness (Darabont et al., 2015), which may result in an increased penetrance of excess pulsatility into the cerebrovascular, causing endothelial injury and heightening the oxidative and inflammatory state in the brain (Iulita et al., 2018). In addition, systolic BPV was positively associated with the burden of cerebral small vessel lesions (de Heus et al., 2020). The burden of cerebral small vessel lesions in the basal ganglia was positively correlated with the severity of cognitive impairments in PD (Chen et al., 2021). Accounting for specific lesions, higher systolic BPV was related to a higher burden of white matter hyperintensity (Tully et al., 2018). Meta‐analysis containing 2360 patients with PD from 24 studies reported that patients with dementia had a higher burden of white matter hyperintensity than patients with NC (Butt et al., 2021).

We found that PD‐MCI and PD‐NC groups had comparable first year of VIM, which was inconsistent with the previous study (Kwon et al., 2016). The shorter stage of disease duration in our study may contribute to the different results (Kwon et al., 2016). Our findings suggested that during the early stages of PD, the neurodegenerative pathology might be too subtle to impact VIM significantly. The different degrees of neurodegeneration, particularly evident with aging and PD progression, could explain why VIM predicted cognitive decline in PD‐MCI but not in PD‐NC (Halliday et al., 2014). Elevated BPV was associated with increased levels of phosphorylated tau and total tau, as well as decreased levels of beta‐amyloid in the CSF of both cognitively unimpaired and mildly impaired older adults (Sible et al., 2022). However, we failed to find any association between VIM and the CSF and serum biomarkers which could be contributed by the limited follow‐up times of biomarkers.

There were several strengths of our study. We conducted sensitivity analyses to exclude the influence of the anti‐Parkinson drugs and diagnostic criteria to confirm the stability of our results. The current study had some limitations. First, BP was assessed in the supine position instead of the sitting position described in previous studies (Böhm et al., 2015; Ernst et al., 2021; Lattanzi et al., 2015; Qin et al., 2016; Rouch et al., 2020). Considering the difference between the supine BP value and sitting BP value, we used VIM as the primary indicator of BPV, whose value was independent of the mean BP value. Second, the impact of nocturnal BP was not assessed in our study. Future studies were needed to explore whether supine BPV was linked to abnormal nocturnal BP. In addition, only MoCA was used for the cognitive test, which restricted the exploration of the relationship between the specific cognitive region and the BPV. Using the insensitive single cognitive test scale may miss the minor cognitive decline and decline in specific cognitive regions during follow‐up. The change of scores of MoCA in PD‐NC may be too mild to provide the necessary dynamic range for the current analysis.

## 5 CONCLUSIONS AND IMPLICATIONS

In conclusion, this longitudinal study included 297 patients with early PD, exploring the association between cognition and visit‐to‐visit supine BPV. Higher systolic VIM was found to be related to a greater rate of decline in MoCA scores in patients with PD‐MCI.

## AUTHOR CONTRIBUTIONS

**Yi Xiao**: Methodology; conceptualization; formal analysis; writing—original draft; writing—review and editing. **Tianmi Yang**: Writing—review and editing; formal analysis. **Lingyu Zhang**: Writing—review and editing. **Qianqian Wei**: Writing—review and editing. **Ruwei Ou**: Writing—review and editing. **Yanbing Hou**: Writing—review and editing. **Kuncheng Liu**: Writing—review and editing. **Junyu Lin**: Writing—review and editing. **Qirui Jiang**: Writing—review and editing. **Huifang Shang**: Writing—review and editing; methodology.

## CONFLICT OF INTEREST STATEMENT

There were no financial conflicts of interest to disclose.

### FUNDING INFORMATION

No specific funding was received for this work.

### PEER REVIEW

The peer review history for this article is available at https://publons.com/publon/10.1002/brb3.3319.

## CONSENT FOR PUBLICATION

The authors confirmed that this manuscript was approved for publication by the PPMI Data and Publications Committee.

## Supporting information

Table S1 VIM between groups with different blood pressures.
Table S2 Average follow‐up times (from first year to the following times) in different groups.

## Data Availability

All the data can be downloaded from the PPMI database (www.ppmi‐info.org/access‐data‐specimens/download‐data) with the consent of PPMI Data and Publications Committee.
