# Supplementary material for: Association between the blood pressure variability and cognitive decline in Parkinson's disease
Source: Brain Behav. 2023 Nov 15;13(12):e3319. doi: 10.1002/brb3.3319 (PMC10726805; doi:10.1002/brb3.3319)
Supplement: Supplementary file 1 — Table S1 VIM between groups with different blood pressures. Table S2 Average follow‐up times (from first year to the following times) in different groups. [file BRB3-13-e3319-s001.docx]

**Supplementary Methods**

**PPMI cohort**

PPMI is a prospective, observational, multicenter study focused on PD in the early stage. The brief inclusion criteria of the PPMI cohort were: 1) diagnosed as PD within two years; 2) untreated for PD; 3) having dopamine transporter deficit proved by dopamine transporter imaging. The details of this cohort were described in the former articles(Parkinson Progression Marker, 2011).

**Evaluation**

Demographic features including age, sex, disease duration, education level, body mass index, comprehensive cognition assessment and medical history were recorded. Comorbidities were also recorded, including hypertension, heart disease, type 2 diabetes mellitus, and hyperlipidemia.

Blood pressure and MDS-UPDRS were assessed at baseline, every 3-month for the 1^st^ year, and every 6-month in the following years. MoCA, Rapid Eye Movement Sleep Behavior Disorder Screening Questionnaire, and Geriatric Depression Scale were conducted at baseline and every year during the follow-up.

**Calculation of the blood pressure indicator**

Standard deviation and coefficient of variation were the most popular indicators of blood pressure variety. However, previous studies found that the two indicators were strongly correlated to the mean values.(Howard & Rothwell, 2009; Rothwell et al., 2010) Variation independent of the mean (VIM) was proposed as an alternative measurement of the blood pressure variety, eliminating the effect of mean value of blood pressure.(Asayama et al., 2015; Rothwell et al., 2010) The calculation method of VIM was listed as follows.

$$VIM=(SD*{population mean}^{x}/{mean}^{x})$$

The $\mathrm{SD}$ was the standard deviation of the blood pressure records of the patient. The $\mathrm{mean}$ was the mean value of the blood pressure records of the patient. The $x$ was obtained by fitting a curve through a plot of $\mathrm{SD}$ against the $\mathrm{mea}n$ using the model listed below.

$$SD={a*mean}^{x}$$

**Sensitivity analysis**

A drop in blood pressure was observed after taking levodopa (Noack, Schroeder, Heusser, & Lipp, 2014), and the use of dopamine agonists, amantadine, and monoamine oxidase-B inhibitors may have side effects such as causing hallucinations and contributing to cognitive decline. The first sensitivity analysis was implemented by including the time-varying levodopa equivalent drug dose, use of dopamine agonists, amantadine, and monoamine oxidase-B inhibitors in the models.

In addition, the total correction of MoCA to detect the PD-MCI was around 64%,(Litvan et al., 2012) so we used alternative diagnosis criteria to define the PD-MCI as the second sensitivity analysis. Patients were diagnosed with PD-MCI if two or more scores of the Hopkins Verbal Learning Test immediate/total recall, Hopkins Verbal Learning Test recognition discrimination, Benton Judgment of Line Orientation, Letter-Number Sequencing, Semantic fluency Test, and Symbol-Digit Modalities Test were more than 1.5 standard deviations below normal, and without functional impairment due to cognitive impairment.(Litvan et al., 2012)

Asayama, K., Wei, F. F., Liu, Y. P., Hara, A., Gu, Y. M., Schutte, R., . . . Staessen, J. A. (2015). Does blood pressure variability contribute to risk stratification? Methodological issues and a review of outcome studies based on home blood pressure. *Hypertens Res, 38*(2), 97-101. doi:10.1038/hr.2014.153

Howard, S. C., & Rothwell, P. M. (2009). Reproducibility of measures of visit-to-visit variability in blood pressure after transient ischaemic attack or minor stroke. *Cerebrovasc Dis, 28*(4), 331-340. doi:10.1159/000229551

Litvan, I., Goldman, J. G., Troster, A. I., Schmand, B. A., Weintraub, D., Petersen, R. C., . . . Emre, M. (2012). Diagnostic criteria for mild cognitive impairment in Parkinson's disease: Movement Disorder Society Task Force guidelines. *Mov Disord, 27*(3), 349-356. doi:10.1002/mds.24893

Noack, C., Schroeder, C., Heusser, K., & Lipp, A. (2014). Cardiovascular effects of levodopa in Parkinson's disease. *Parkinsonism Relat Disord, 20*(8), 815-818. doi:10.1016/j.parkreldis.2014.04.007

Parkinson Progression Marker, I. (2011). The Parkinson Progression Marker Initiative (PPMI). *Prog Neurobiol, 95*(4), 629-635. doi:10.1016/j.pneurobio.2011.09.005

Rothwell, P. M., Howard, S. C., Dolan, E., O'Brien, E., Dobson, J. E., Dahlof, B., . . . Poulter, N. R. (2010). Prognostic significance of visit-to-visit variability, maximum systolic blood pressure, and episodic hypertension. *Lancet, 375*(9718), 895-905. doi:10.1016/S0140-6736(10)60308-X

Supplementary table 1 VIM between groups with different blood pressure characteristics

|  | N | Systolic VIM | P* | Diastolic VIM | P* |
| --- | --- | --- | --- | --- | --- |
| With orthostatic hypotension | 49 | 8.78 (6.26–11.35) | 0.240 | 5.00 (3.82–7.75) | 0.799 |
| Without orthostatic hypotension | 248 | 7.99 (5.58–10.48) |  | 5.57 (3.69–7.3) |  |
| With hypertension | 119 | 8.42 (5.85–10.9) | 0.441 | 5.61 (3.88–7.72) | 0.692 |
| Without hypertension | 178 | 7.83 (5.54–10.38) |  | 5.33 (3.63–7.04) |  |
| With supine hypertension | 81 | 8.4 (5.87–11.37) | 0.410 | 5.52 (3.71–7.88) | 0.365 |
| Without supine hypertension | 216 | 8.01 (5.69–10.37) |  | 5.38 (3.87–7.22) |  |

*Significant at level 0.05.

VIM: Variation independent of mean.

Supplementary table 2 Average follow-up times (from 1^st^ year to the following times) in different groups

| Variables | Parkinsons’s disease MCI | Parkinsons’s disease with NC |
| --- | --- | --- |
| MoCA | 6.30 | 6.33 |
| CSF Aβ42 | 2.08 | 2.07 |
| CSF Aβ | 1.56 | 1.68 |
| Serum Nfl | 3.25 | 3.12 |
| CSF NfL | 1.88 | 1.79 |
| CSF ptau-181 | 3.60 | 3.70 |
| CSF total tau | 3.60 | 3.70 |
| CSF α-synuclein | 2.08 | 2.07 |
| CSF GFAP | 1.88 | 1.79 |

Abbreviation: MCI: mild cognitive impairment; NC: normal cognition; MoCA: Montreal Cognitive Assessment. VIM: variation independent of the mean; CSF: cerebrospinal fluid; Aβ42: amyloid-β1-42; Aβ: β-amyloid; p-tau181: tau phosphorylated at the threonine 181 position; NfL: neurofilament light protein; GFAP: glial fibrillary acidic protein.
